# Cinnamon Oil Encapsulated with Silica Nanoparticles: Chemical Characterization and Evaluation of Insecticidal Activity Against the Rice Moth, *Corcyra cephalonica*

**DOI:** 10.1007/s13744-023-01037-1

**Published:** 2023-03-13

**Authors:** Radwa G. Attia, Mostafa M. H. Khalil, Mohamed A. Hussein, Hoda M. Abdel Fattah, Salwa A. Rizk, Shireen A. M. Ma’moun

**Affiliations:** 1 Entomology Department, Faculty of Science, Ain Shams University, Cairo, Egypt; 2 Chemistry Department, Faculty of Science, Ain Shams University, Cairo, Egypt; 3 National Center for Radiation Research and Technology, Atomic Energy Authority, Cairo, Egypt

**Keywords:** Encapsulation with nanoparticles, Cinnamon essential oil, Peppermint oil, Silica gel, Mesoporous silica nanoparticles, *Corcyra cephalonica*

## Abstract

Cinnamon (*Cinnamomum zeylanicum* Blume) essential oil has vast potential as an antimicrobial but is limited by its volatility and rapid degradation. To decrease its volatility and prolong the efficacy of the biocide, cinnamon essential oil was encapsulated into mesoporous silica nanoparticles (MSNs). The characterization of MSNs and cinnamon oil encapsulated with silica nanoparticles (CESNs) was estimated. Additionally, their insecticidal activity against the rice moth *Corcyra cephalonica* (Stainton) larvae was evaluated. The MSN surface area decreased from 893.6 to 720 m^2^ g^−1^ and the pore volume also decreased from 0.824 to 0.7275 cc/g after loading with cinnamon oil. X-ray diffraction, Fourier transform infrared spectroscopy (FTIR), energy-dispersive X-ray spectroscopy (EDX), and N2 sorption by Brunauer–Emmett–Teller (BET) confirmed the successful formation and evolution of the synthesized MSNs and CESN structures. The surface characteristics of MSNs and CESNs were analyzed by scanning and transmission electron microscopy. Compared with the sub-lethal activity values, the order of toxicity after 6 days of exposure was MSNs ˃ CESN ˃ cinnamon oil ˃ silica gel ˃ peppermint oil. The efficacy of CESNs gradually increases its toxicity more than MSN after the 9th day of exposure.

## Introduction

The rice moth *Corcyra cephalonica* Stainton (Lepidoptera: Pyralidae) is one of the most serious pests of grains and stored products. This insect infests rice, cashew nuts, date palm, raisins, corn, cocoa, dried fruits, almonds, chocolates, biscuits, oilcakes, coffee, and other seeds (Jagadish et al. 2009). Damage is mostly caused by larvae; during storage, the larvae spin tough silken fibers that web together the kernels, frass, and exuviae. Webbing and insect cadavers form masses that cause troubles in mills because they block flour tubes and stop milling machines, and they also cause quantitative and qualitative losses and reducing the germinability of seed stocks (Senguttuvan et al. 1995). Insects also induce changes in the storage environment that may cause warm and moisture, which are suitable for the development of storage fungi, thereby causing further losses. Therefore, postharvest studies should be prioritized.

The current methods for managing stored grain pests depend heavily on chemical pesticides. However, repeated use of these pesticides has led to the appearance of genetically resistant pest species (Yao et al. 2019; Attia et al. 2020a, b). Unfortunately, they cause contamination of food with toxic residues, thereby increasing application costs, environmental pollution, and health hazards (Dubey et al. 2007).

Silica nanoparticles have been suggested as potential candidates for increasing the control over a range of agricultural pests (Barik et al. 2008) and are manufactured by various methods, and they all have the common formula SiO_2_. The commercial use of amorphous silica is approved by the United States Department of Agriculture (USDA) (Stathers et al. 2004) and registered as a human food additive. The application of silicon in the field reduced pest infestation and increased crop tolerance such as susceptible rice and wheat (El-Samahy 2002; Basagli et al. 2003; and Ecole and Sampaio 2004). Si accumulates in the plant epidermal cells. However, it increases the hardness of plant tissues and consequently affecting the oral parts of phytophagous insects and reducing food intake and when Si concentration increases the change subsequently increases (Strömberg et al. 2016 and Jeer et al. 2017). Si-enriched tissues also show a reduced digestibility and palatability, leading to a decrease in insect’s growth rate (Massey and Hartley 2009 and Frew et al. 2016). Si also affects the feeding behavior of phytophagous insects, as observed in *Spodoptera exempta* (Walker) and *Schistocerca gregaria* (Forskal) that avert plants treated with Si (Massey et al. 2006).

Several studies have focused on the potential use of essential oils (EOs) in the biological control of different insect pests since some are selective, safe to apply, biodegraded to nontoxic products, potentially less expensive, and have few effects on nontarget organisms and the environment (Isman 2000; Kim et al. 2010 and Eesiah et al. 2022). Many recent studies have been performed on the toxicity and biological effects of certain natural oils on different insect species, giving promising results (Mohamed et al. 2020; Attia et al. 2020a, b; Elsayed et al. 2022 and Sayed et al. 2022). Although essential oils have promising properties, the problems related to their poor water solubility, volatility, evaporation under direct exposure to heat, light, pressure or oxygen, and potential for oxidation reduces their effectiveness in the field and storage life. Hence, these problems must be resolved before EOs are used as alternative pest control means (Moretti et al. 2002).

Nanoencapsulation of essential oils has the following advantages: they can be easily ingested by the target organisms, are more active than normal pesticides, are ecofriendly and biodegradable, and do not lead to the development of resistance, as observed for crude plant-based nanopesticides (Kumar et al. 2022); moreover, only small quantities are required, they have a long shelf life, and they cause less pollution (Kumar et al. 2019; Lade et al. 2019 and Deka et al. 2021). Finally, the controlled release of active substance allows for the more effective use of the oils over a given time interval (Shrankhla 2014).

The purpose of the present study was to evaluate the effect of cinnamon essential oil encapsulated with mesoporous silica nanoparticles and the impact of encapsulation components individually (MSNs, silica gel, cinnamon oil compared with peppermint oil as an alternative essential oil) on the stored product pest *C. cephalonica* under laboratory conditions.

## Materials and methods

### Insect culture

The rice moth *C. cephalonica* used in this study was obtained from a laboratory susceptible strain maintained for several generations in the National Center for Radiation Research and Technology (NCRRT), Atomic Energy Authority, Nasr City, Cairo, Egypt.

Newly emerged adults were allowed to mate and oviposit in inverted jars with screen tops. The eggs were collected with wire mesh and then transferred for breeding in jars containing sterilized whole wheat flour mixed with yeast at a ratio of 40-g to 1-kg flour. Larvae developed in 33–55 days. Full-grown larvae were allowed to pupate, and pupae were placed singly in a glass vial until adult emergence. The two sexes were segregated in the adult stage. All the stock cultures were maintained under controlled conditions of 26 ± 1 °C, 70 ± 5% RH, and photoperiod of 16:8-h light/dark cycle.

### Chemicals and essential oils used

Cinnamon and peppermint essential oils were purchased from the National Research Centre, Dokki: Giza, Egypt.

Mesoporous silica nanoparticles, cinnamon oil encapsulated with silica nanoparticles, and silica gel were synthesized locally in Chemistry and Entomology Departments-Faculty of Science-Ain Shams University.

### Synthesis of mesoporous silica nanoparticles

According to the protocol described by (Shahat and Trupp 2017) with some modifications, mesoporous silica nanoparticles (MSNs) were synthesized by hydrolysis of tetraethyl orthosilicate (TEOS) obtained from Sigma–Aldrich as a liquid in a mixed solvent of water, acetone, diethyl ether, and ammonium hydroxide using the cationic surfactant cetyl trimethylammonium bromide (CTAB) at room temperature.

CTAB (1 mg) was dissolved in 200 ml of deionized water by stirring for 30 min. After that, 80 ml of acetone was added to the solution and stirred for 15 min. Then, 40 ml of diethyl ether was added, and the solution was left to stir for 30 min. Typically, 5 ml of TEOS was added drop by drop to the solution and stirred for 30 min, followed by the addition of 3 ml ammonium hydroxide solution (25%). The resulting gel solution was stirred for 24 h at room temperature. The resulting silica/CTAB solid particles were filtered and washed with deionized water and allowed to dry for 3 h at 80 °C in a dryer. Then, the obtained white mesoporous silica nanoparticles were calcinated to remove the templating agent by gradually increasing the temperature from room temperature to 550 °C for 8 h.

### Preparation of cinnamon essential oil encapsulation

Cinnamon essential oil encapsulation was prepared according to the method of Dohare et al. (2014). Five hundred milligrams of mesoporous silica nanoparticles was placed in a capped conical tube, and 5 ml of Triton X-100 was added to increase the porosity. Then, 25 ml of cinnamon oil was added to the solution. The obtained solution was continuously stirred for four hours on a magnetic stirrer. Then, the suspension was centrifuged for 15 min at 10,000 rpm, and the obtained precipitate was dried at room temperature in a Petri dish.

### Characterization and analysis of nanoparticles

#### Scanning and transmission electron microscopy

The morphology of mesoporous silica nanoparticles (MSNs) and cinnamon oil encapsulated with silica nanoparticles (CESN) was analyzed by scanning electron microscopy. Particle size of MSNs was determined by transmission electron microscope (Model JEOL JSM 5200, Japan). Sample preparation was carried out by immersion in glutaraldehyde buffer (0.1 M) for 2 h at 4 °C (pH = 7.3), post-fixation by osmium tetraoxide (0.1 M) for 1 h at 4 °C, followed by dehydrating the samples by 30, 50, and 70% ethyl alcohol consecutively for 2 min for each, and remained in 100% ethyl alcohol for 30 min at 4 °C. Finally, the samples were mounted on a piece of adhesive paper and gold coated using a vacuum coater (Sputter Coater, Japan).

####  X-ray diffraction (XRD)

XRD was carried for determining the atomic and molecular structure of MSNs and CESN. Measurements were performed on Shimadzu X-ray. About 1 g of each sample was mounted directly on the metal holder and analyzed. The X-rays were generated at 30 mA current and 40 kV voltage equipped with a Cu target and CuKα (*λ* = 0.15406 nm) were selected. Samples were scanned in the range of diffraction angle (2θ) 3° to 90° at 2°/min scanning rate.

#### Fourier transform infrared spectroscopy (FTIR)

FTIR was performed on a Thermo Scientific Nicolet 380 FTIR spectrometer to obtain MSN and CESN chemical properties. Samples were grinded up with potassium bromide (KBr) for transmission measurements. Then, they were subjected to pressure and form a thin disk that is IR transparent. For making disks, the grinded samples were put into a pellet-forming die. A hydraulic press is used to press the sample with several tons of force. The synthetic sample–KBr pellet is then analyzed in transmission mode. FTIR spectra of the MSN and the CESN were acquired in the range of 400–4000 cm^−1^.

#### Energy-dispersive X-ray spectroscopy (EDX)

The morphological and the compositional properties of the silica nanoparticles were monitored by EDX spectroscopy (JEOL Type JSM—6510 LA equipment) which is coupled with electron microscope.

#### Brunauer–Emmett–Teller (BET)

The N_2_ sorption (adsorption/desorption) isotherm measurements of MSN was done by BET using Nova Station B at the National Center for Radiation Research and Technology (NCRRT), Atomic Energy Authority, Nasr City, Cairo, Egypt. For CENS, N2-sorption (BET) was done in Japan. Samples first were degassed in vacuum stations under high temperature 473 K for 10 h then subjected to liquid nitrogen in the analysis station.

### Bioassay test

The insecticidal activity of cinnamon and peppermint oils was evaluated against 6^th^ instar larvae of *C. cephalonica* by the contact method. Four concentrations (25%, 50%, 75% and 100%) equivalent to 125, 250, 375, and 500 mg/cm^2^ of each oil were prepared by diluting pure oil with distilled water and adding Tween 80 at 5 wt%. Filter papers were impregnated with each concentration, placed inside plastic boxes, and left for 24 h to dry. Then, larvae were inoculated into the plastic boxes. The control experiment contained Tween 80 only.

Additionally, a bioassay test was carried out to evaluate the toxicity of mesoporous silica nanoparticles, cinnamon oil encapsulated with silica nanoparticles and silica gel. Four concentrations (15 mg, 30 mg, 60 mg, and 90 mg) were used. Each concentration of each compound was mixed with 2 g of crushed wheat grains for oral feeding larvae of the treated samples and 2 g of crushed wheat grains for the control. The containers were manually shaken for approximately 60 s to obtain an equal distribution of each compound. The plastic containers were kept for 24 h at room temperature before inserting *C. cephalonica* larvae.

Each concentration and untreated test was administered to four groups of 10 larvae. The percent larval mortality was calculated daily for different concentrations and for control larvae. 6th instar larvae were tested after 6 days of treatment with these compounds.

### Statistical analysis

The data were analyzed based on one-way analysis of variance (ANOVA) using computer statistical (SPSS) version 16. All levels of statistical significance between the concentrations of compounds and percentage mortality of *C. cephalonica* larvae were determined by the LSD test at the 95% confidence limit.

Computed mortality percentage was plotted versus the corresponding concentration using probitvb6 software program to obtain the toxicity regression line, LT_50_, LC_50_ and lC_95_ values, and slope of the tested compounds.

## Results and discussion

### Characterization of mesoporous silica nanoparticles

#### X-ray diffraction (XRD)

**Fig. 1:**
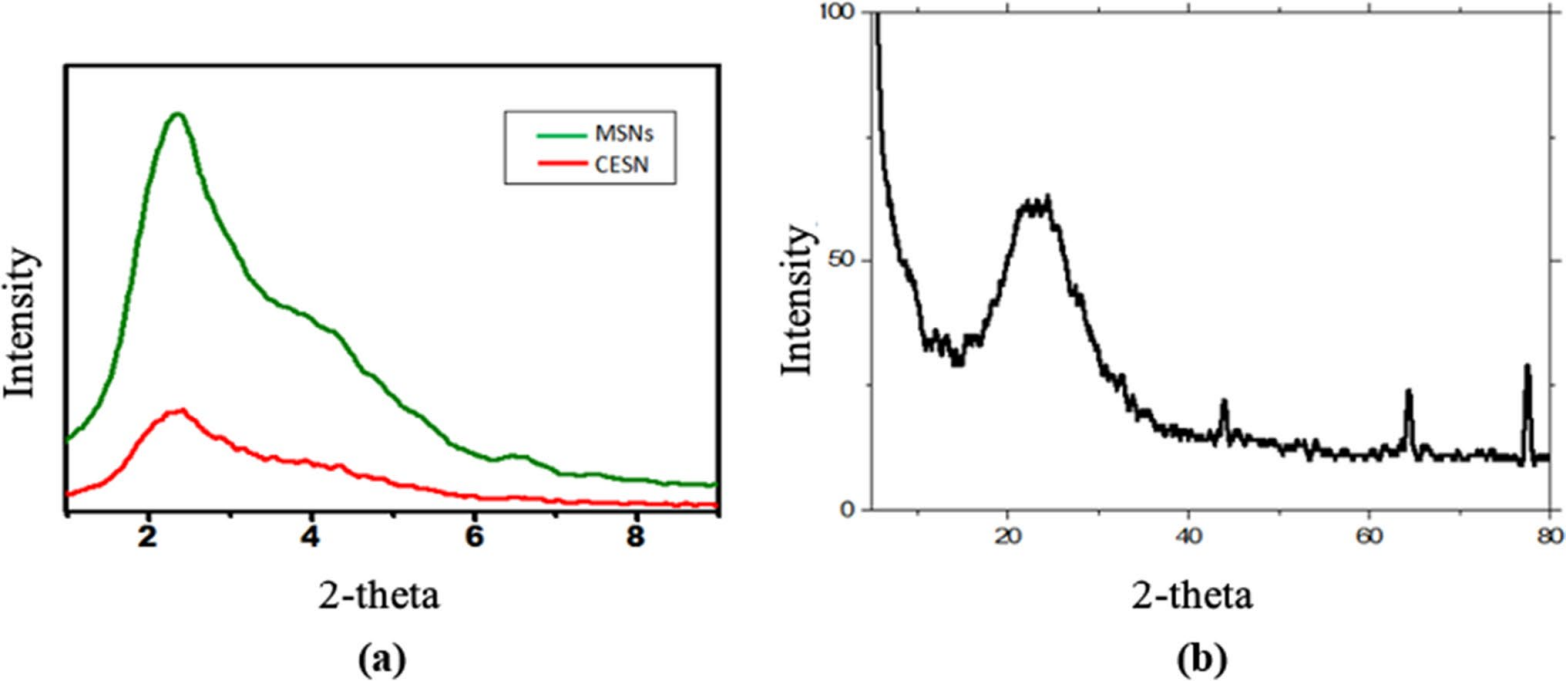
**a** SAXRD of the synthesized mesoporous silica nanoparticles and cinnamon oil encapsulated with silica nanoparticles and **b** WAXRD of mesoporous silica nanoparticles

The results revealed the formation of mesoporous silica with a stable morphology. In Fig. 1, the small angle X-ray diffraction (SAXRD) patterns of fabricated mesoporous silica nanoparticles (Fig. 1a) when impinged with X-rays diffracted at an angle of 2θ showed a strong and clear single diffraction with an angle of 2.2°, indicating the formation of a mesostructure (Elshehy et al. 2017). Moreover, our findings showed that the crystal geometry is hexagonal-like and transitions to a less ordered mesophase (Zhang et al. 2010). Otherwise, the data of wide-angle WXRD data (Fig. 1b) revealed a broad peak at approximately 24 degrees, which confirms the construction of crystallized mesoporous silica. To confirm the successful decoration of the mesoporous silica via cinnamon oil, the designed cinnamon oil encapsulated with silica nanoparticles (CESNs) was examined using SAXRD. Our results showed the same diffraction peaks, which indicate that the high stability of the trapped cinnamon oil inside the mesoporous caves (Fig. 1a) changes the diffraction intensities between the MSN and CESN capsules due to the decrease in the porosity of the MSNs, which indicates that the pores are filled with cinnamon oil.

#### Fourier transform infrared spectroscopy (FTIR) analysis

**Fig. 2:**
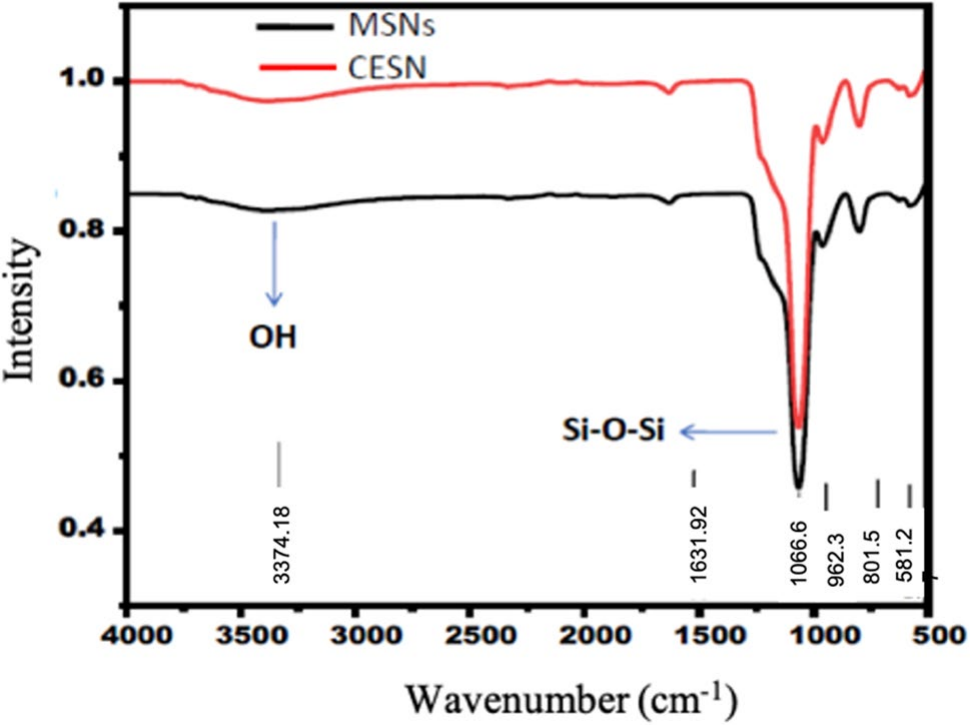
FTIR spectra of mesoporous silica nanoparticles and cinnamon oil encapsulated with silica nanoparticles

To examine the successful formation of MSNs and CESNs, the chemical structure of the synthesized nanoparticles was analyzed by FTIR. The spectra exhibited a number of characteristic spectral bands; the peak at 1066.63 cm^−1^ of the Si–O–Si stretching vibration band, which is the specific band of mesoporous silica nanoparticles (Liu et al. 2012 and Salami-Kalajahi et al. 2012), indicates successful conversion into SiO_2_ (Fig. 2). Furthermore, the broad stretching vibration band at approximately 3374.18 cm^−1^ can be attributed to the (OH) group (Liu et al. 2012 and Salami-Kalajahi et al. 2012), indicating the formation of the silanol group Si–OH. Moreover, our result indicates the functionality of the designed mesoporous silica with active multiple phenol groups to directly interact with the immobilized material. The FTIR spectrum of cinnamon oil encapsulated with silica nanoparticles (Fig. 2) retained most of the major peaks of the silica nanoparticles.

#### Energy-dispersive X-ray spectroscopy (EDX) analysis

**Fig. 3:**
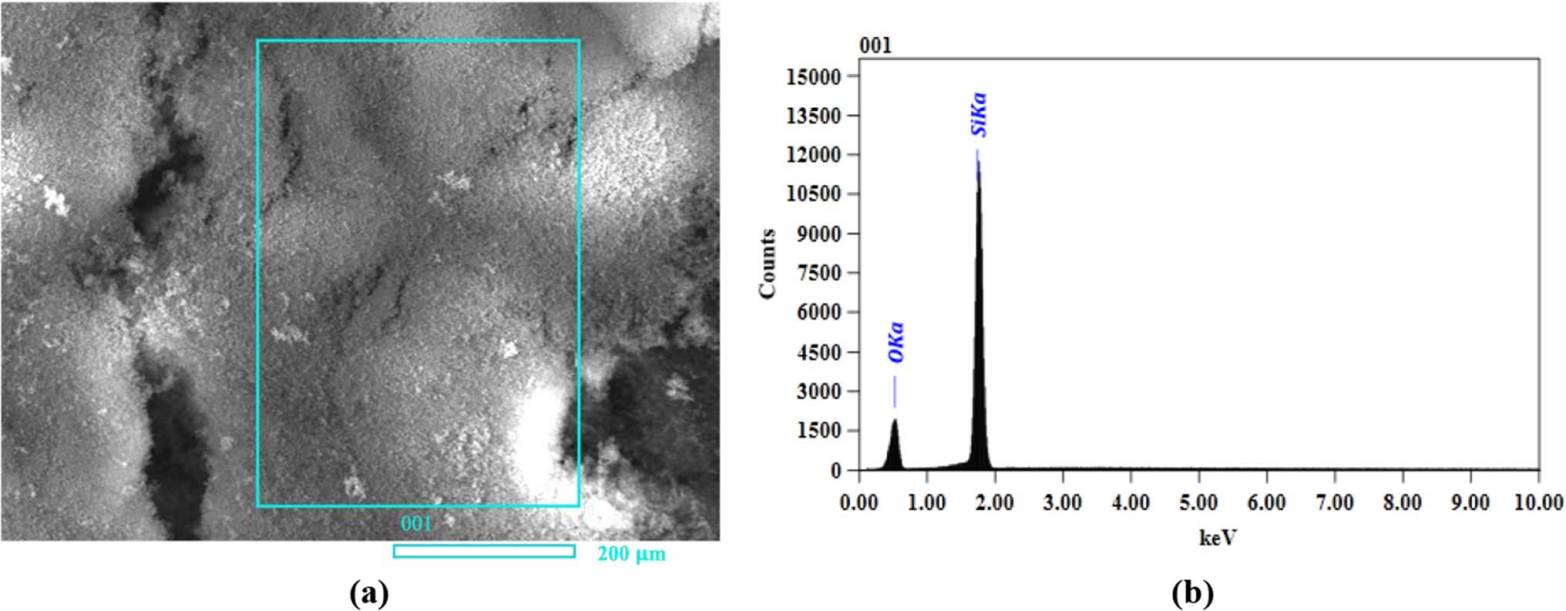
**a** Scanning electron microscope image of synthesized mesoporous silica nanoparticles. **b** EDX spectra of synthesized mesoporous silica nanoparticles

Energy-dispersive X-ray spectra were obtained coupled with SEM images (Fig. 3a) to confirm successful MSN formation. The results indicated that the synthesized silica nanoparticles have a major component of SiO_2_; hence, oxygen and silicon were the major contributors to the atomic weight percentage. The results are presented as the EDX spectra. The weight percentage of the synthesized mesoporous silica nanoparticles was 42.93% silicon and 57.07% oxygen (Table 1). Mapping of fabricated MSNs (Fig. 3b) indicates the elemental composition. Mapping is an appropriate process to indicate the distribution of each element clearly. However, those results were not consistent with the results of Nasreen et al. (2018), who found that the percentage of silica and oxygen in synthesized MSNs was 25.63 and 74.37%, respectively.

**Table 1:** Percentage of mass and atoms of mesoporous silica nanoparticles

Elements	Mass %	Atom %
O K	43.10	57.07
Si K	56.90	42.93

### Brunauer–Emmett–Teller (BET) surface area analysis for synthesized MSNs and CESNs

**Fig. 4:**
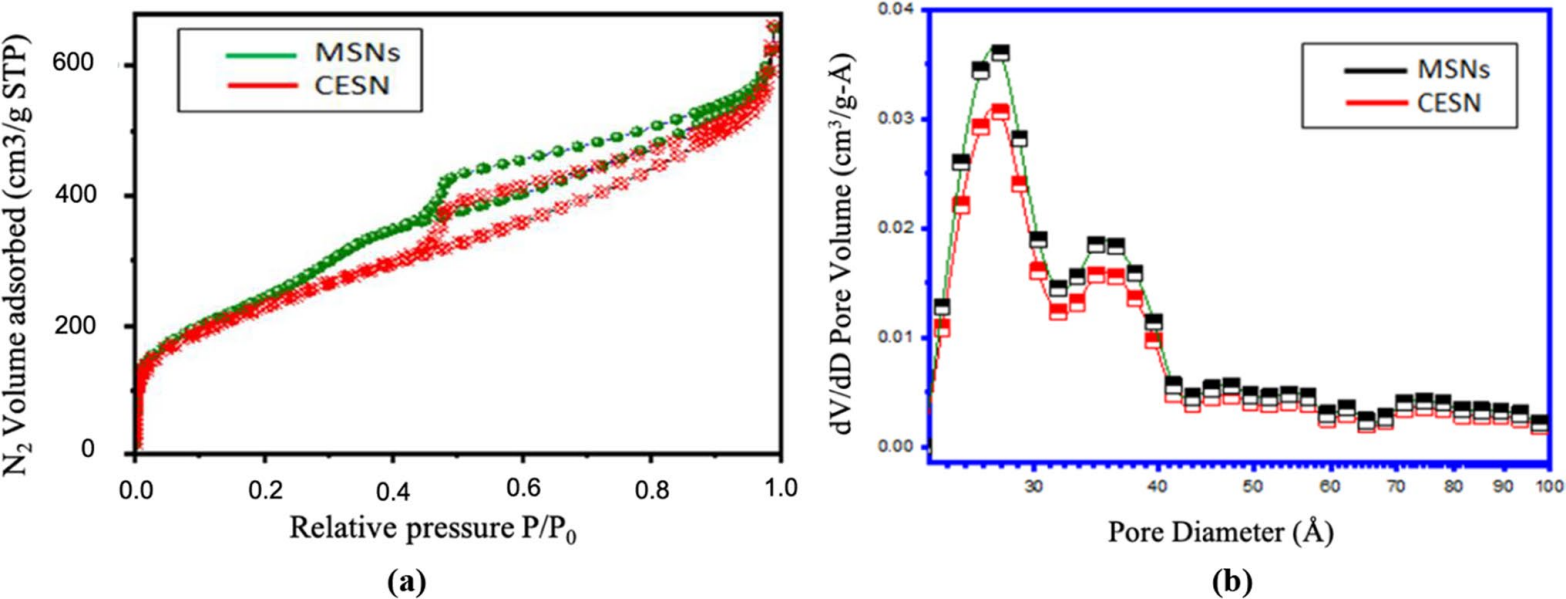
**a** N_2_ adsorption/desorption isotherm of mesoporous silica nanoparticles. **b** Pore size distribution of mesoporous silica nanoparticles and cinnamon oil encapsulated with silica nanoparticles

The N_2_ adsorption/desorption isotherm measurements for the SiO_2_ samples were investigated (Fig. 4a) as described by El-Safty et al. (2015). The resulting isotherms are of type IV, which is characteristic of mesoporous materials with large mesopores. An isotherm of the hysteresis loop characterized by a smooth increase in the adsorption branch and a steep desorption branch as a result of capillary condensation of N_2_ gas in the mesopores. Figure 4 b shows a broad distribution of pore sizes in the range of 11.88 nm calculated by using BJH analysis. Additionally, the pore volume of MSNs (0.824 cc/g) is higher than that of CESNs (0.7275 cc/g). The BET surface area value changed from unloaded MSNs (893.6 m^2^ g^−1^) to MSNs loaded with cinnamon oil (720 m^2^ g^−1^). The decrease in pore volume and surface area ensured that the encapsulation of cinnamon oil molecules into MSNs occurred. This result was consistent with the yolk-like structure shown in the SEM profiles and confirms the successive encapsulation of cinnamon oil with silica nanoparticles. The results of the surface area analysis were consistent with those obtained by Yang et al. (2016), who reported that the resulting isotherms exhibited type IV, which is characteristic of mesoporous materials with highly uniform cylindrical pore mesopores. The present findings were inconsistent with Yuan et al. (2008), who showed that for the isotherms of N_2_ adsorption/desorption of silica nanoparticle-coated organic pigment particles, the slopes of the curves were very small, indicating that very few small pores existed on the surfaces of organic pigment particles.

### Scanning and transmission electron microscopy

**Fig. 5:**
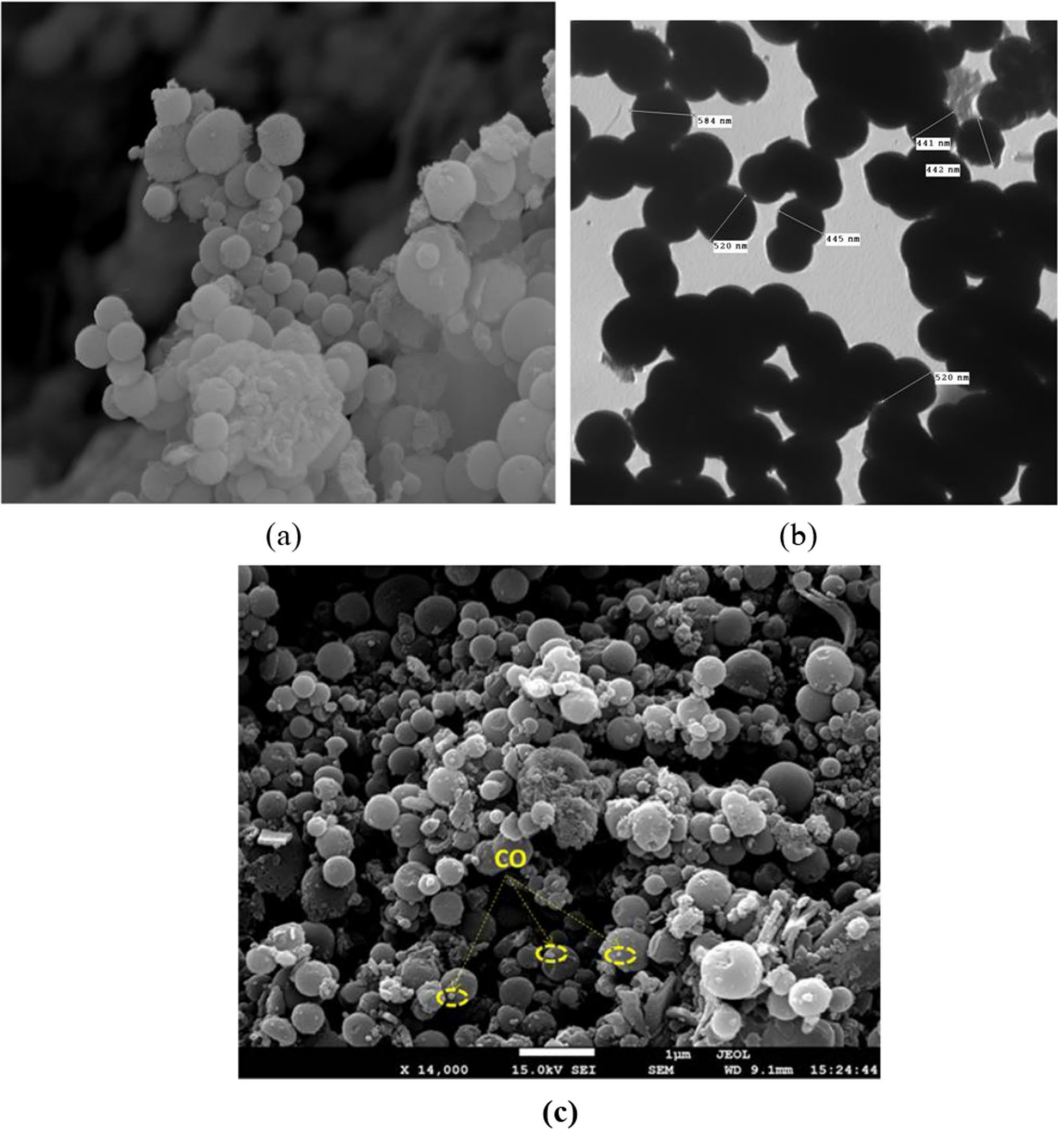
Electron micrograph of the separated spherical arrangements of mesoporous silica nanoparticles with a particle size of approximately 500 nm. **a** Scanning electron microscope (25,000 ×). Scale bar: 1 μm. **b** Transmission electron microscope (1000 ×). Scale bar: 1 μm. **c** Scanning electron microscope of cinnamon oil encapsulated with silica nanoparticles showing decoration of cinnamon oil (CO) in the mesoporous cavities of silica nanoparticles (14,000 ×). Scale bar: 1 μm

The morphological features, including the size and shape of the fabricated MSNs, were assessed under scanning and transmission electron microscopy, as shown in Fig. 5a and b. The synthesized mesoporous silica was in a separated spherical arrangement with an average particle size of approximately 500 nm, homogenous and uniform, which confirmed the successful fabrication of MSNs.

The scanning electron microscope (SEM) image of CESNs shown in Fig. 5c revealed the formation of a yolk-like structure on the mesoporous silica nanoparticle surface loaded with cinnamon oil particles, which was completely consistent with the XRD patterns.

Dohare et al. (2014); Ebadollahi et al. (2017); Cadena et al. (2018); Zea et al. (2018); and Melendez-Rodriguez et al. (2019) also reported that the average particle size of fabricated MSNs was more than 100 nm using a transmission electron microscope (TEM). Owing to their small size, large surface area, and pore volume, silica nanoparticles are a good candidate for the controlled release of the active substance and facilitate its delivery to the target site, ultimately reducing the required dose (Sasson et al. 2007).

### Insecticidal activity of MSNs, CESNs, EOs, and silica gel against C. cephalonica

Employing nanoformulations with essential oils could reduce pesticide applications and further contribute to a better ability to control target pests. Here, cinnamon oil encapsulated with silica nanoparticles (CESNs) and mesoporous silica nanoparticles (MSNs) were applied as biopesticides to realize various benefits for nanotechnology applications for pesticides (Galal and El-Samahy 2012).

**Fig. 6:**
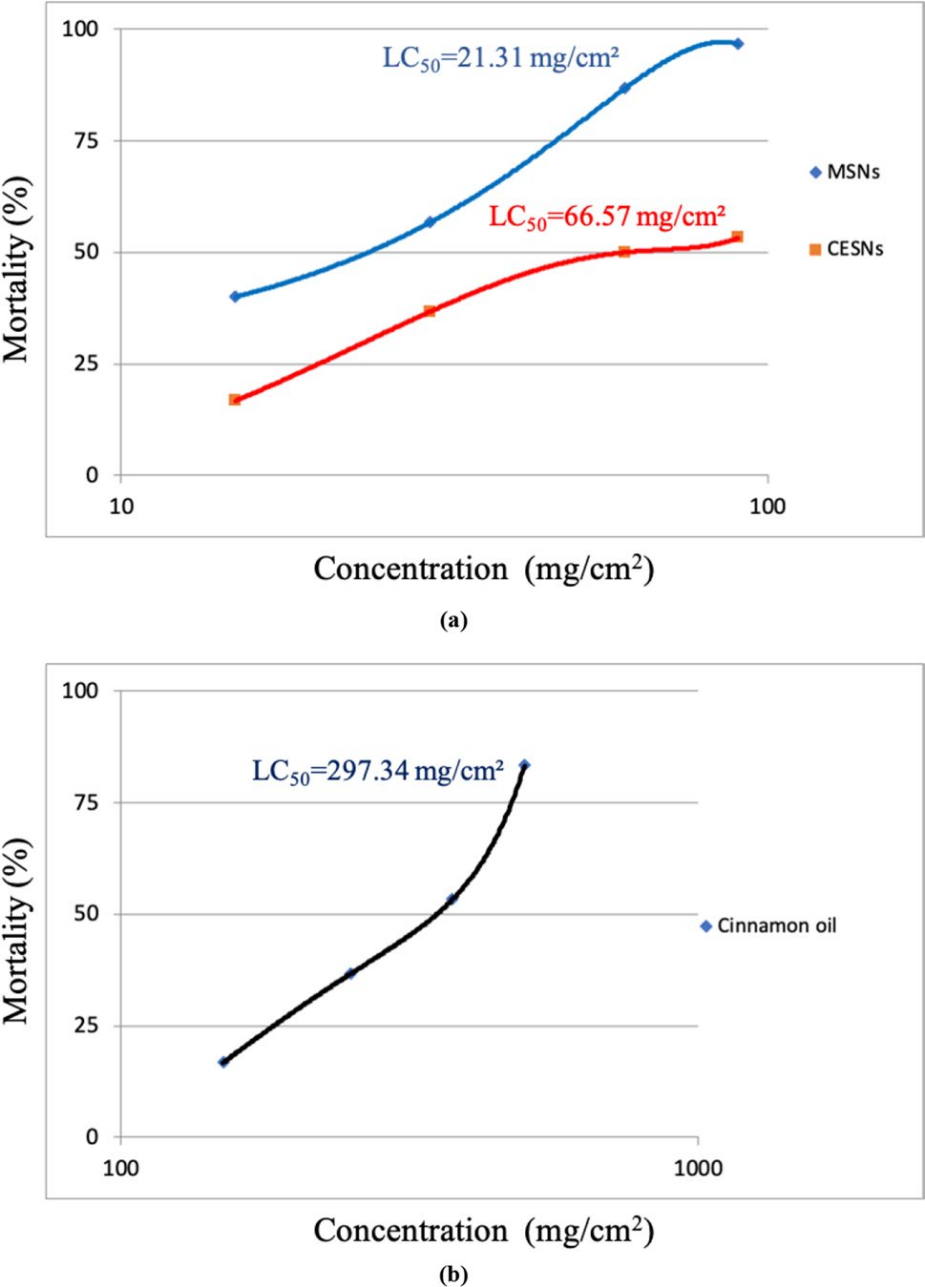
Effect of different concentrations of tested compounds and essential oils on the percent mortality of *Corcyra cephalonica* larvae. **a** Mesoporous silica nanoparticles and cinnamon oil encapsulated by silica nanoparticles. **b** Cinnamon oil

The results are shown in Table 2, where the LC_50_ values of MSNs, CESNs, and cinnamon oil were 21.31 mg, 66.57 mg, and 59.47% (297.34 mg), respectively, and the LC_95_ for the same compounds were 96.78 mg, 1169.08 mg, and 213.263% (1066.32 mg), respectively. For silica gel and peppermint oil, the mortality levels were not sufficient for an adequate LC50 estimation. The maximum mortality achieved were 26.7 and 30% for silica gel and peppermint oil at the highest concentration used, respectively. The order of toxicity according to the LC_50_ was MSNs ˃ CESNs ˃ cinnamon oil (Fig. 6a and b). The larval mortality rate increased with increasing concentrations of compounds and exposure time.

**Table 2:** Mean percent mortality of *Corcyra cephalonica* larvae, LC_50_ and LC_95_ values, their 95% confidence limits and slope of the tested compounds

Tested compounds	Concs. mg/cm^2^	Mortality (%)	LC_50_ and confidence limits (higher limit-lower limit)	LC_95_ and confidence limits (higher limit-lower limit)	Slope	Chi-square
MSNs	15 30 60 90	40 56.7 86.7 96.7	21.31 (28.36–15.95)	96.78 (157.31–60.16)	2.503169 ± 0.2292743	1.466221
CESNs	15 30 60 90	16.7 36.7 50 53.3	66.57 (110.20–40.64)	1169.08 (9992.59–174.77)	1.321652 ± 0.1718853	0.6402717
Cinnamon oil	125 250 375 500	16.7 36.7 53.3 83.3	297.34 (358.96–246.30)	1066.32 (1837.87–620.40)	2.965721 ± 0.3554672	2.6105

% mortality after 6 days from treatment to tested compounds.

0.5 ml of essential oil equals 500 mg of the same oil

MSNs mesoporous silica nanoparticles, CESN cinnamon oil encapsulated with silica nanoparticles.

The most efficient toxicity was observed under the oral feeding of *C. cephalonica* larvae with MSNs. It caused a decrease in larval survival by 40% mortality at a minimum concentration of 15 mg. After 6 days of exposure, this mortality increased to 56.7, 86.7, and 96.7% at concentrations of 30, 60, and 90 mg, respectively (Table 2). The toxicity of MSNs was recorded previously against various insects (Mesbah et al. 2017; Arumugam et al. 2016; Ziaee and Ganji 2016; El-Samahy et al. 2014; Vani and Brindhaa 2013; Barik et al. 2012; Galal and El-samahy 2012; Rouhani et al. 2012 and Debnath et al. 2011).

**Fig. 7:**
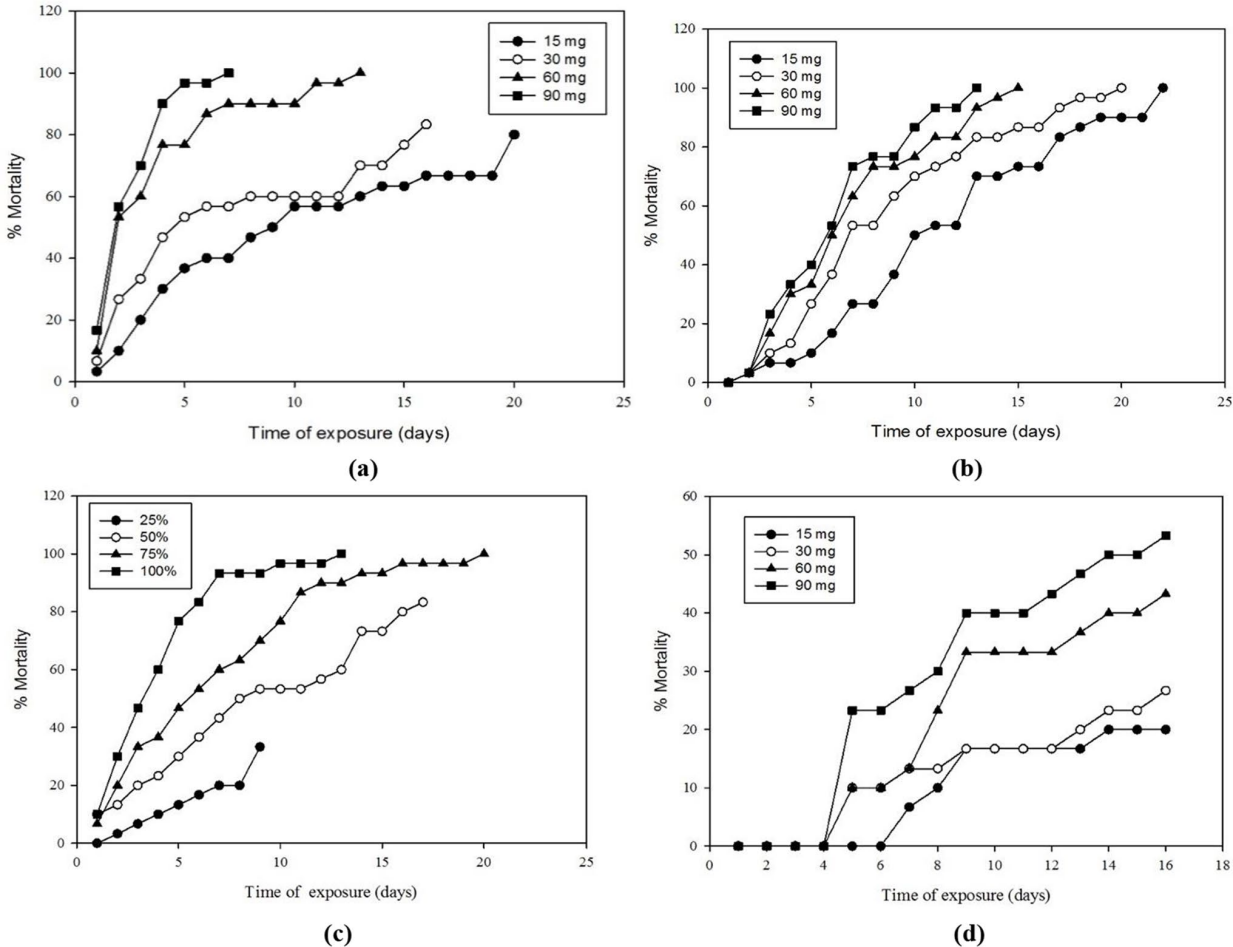
Mortality of *Corcyra cephalonica* larvae through time when exposed to different concentrations of **a** MSNs, **b** CESN, **c** cinnamon oil, **d** silica gel

The second most efficient treatment was CESNs at concentrations of 15, 30, 60, and 90 mg, which caused 16.7, 36.7, 50, and 53.3% mortality, respectively, after 6 days of exposure (Table 2). The toxicity of MSNs showed a higher toxicity relative to the mortality rate in the CESN treatments, as the four concentrations of CESN contain fewer nanosilica particles (approximately one-third) than mesoporous silica nanoparticles alone. This was noticeable at 60 and 90 mg concentrations. In contrast, the efficacy of CESNs became gradually stronger at 15 and 30 mg after 13 and 9 days, respectively, which may be because of the persistent and slow release of the active encapsulated cinnamon oil (Fig. 7a and b).

The third most efficient treatment was cinnamon oil at concentrations of 125, 250, 375, and 500 mg/cm^2^, which caused a decrease in larval survival after 6 days of exposure by 16.7, 36.7, 53.3, and 83.3%, respectively (Table 2). The control efficacy of CESNs was superior to that of free cinnamon oil, as indicated by LC_50_ and LC_95_ concentrations, although the CESN capsules contained approximately 66% trapped cinnamon oil compared to that of the free cinnamon oil. These results are consistent with the results of Campolo et al. (2017) on the tomato borer *Tuta absoluta* (Meyrick); Ebadollahi et al. (2017) on *Tetranychus urticae* Koch (Acari:Tetranychidae); and Gonza´lez et al. (2014) on the German cockroach. In contrast, the efficiency of nanoparticles was lower than that of free garlic essential oil against *Tribolium castaneum* Herbst (Yang et al. 2009).

The fourth treatment was silica gel, which caused no mortality after just four days. After 6 days of exposure, concentrations of 15, 30, 60, and 90 mg caused 6.7, 13.3, 13.3, and 26.7% mortality, respectively (Table 2). Additionally, the results showed that peppermint oil was the least effective, with concentrations of 125, 250, 375, and 500 mg/cm^2^ causing larval reductions of 6.7, 23.3, 23.3, and 30%, respectively, after 6 days of exposure (Table 2). Negligible activity and remarkable mortality delay of peppermint oil were observed relative to cinnamon oil in the *C. cephalonica* control, as indicated by the LC_50_ and LC_95_ concentrations. The obtained results were consistent with that of Lee et al. (2002), who recorded that peppermint oil was less toxic to *Tribolium castaneum* than rosemary, lemon, basil, and lime. In contrast, Choi et al. (2004) revealed that peppermint oil at 4.7 × 10^−3^ μl/ml air was more toxic to *Phytoseiulus persimilis* (Athias-Henriot) adults than the other tested essential oils used at the same dose. Hamza et al. (2016) also reported that peppermint essential oil was the most effective oil against the granary weevil *Sitophilus granarius* (Linnaeus).

Moreover, the present investigation indicated that the efficacy of the tested substances was time and concentration dependent. The larval mortality ratio of *C. cephalonica* treated with different concentrations of MSNs, CESN, cinnamon oil, silica gel, and peppermint oil was recorded after 21, 22, 18, 16, and 23 days of exposure, respectively (Fig. 7a–d). As the time of exposure and the concentration increased, the percentage of larval mortality increased.

As judged by a comparison of sub-lethal time (LT_50_) values of different concentrations of tested substances against 6th instar larvae of *C. cephalonica* (Table 3), the LT_50_ values of the lowest and highest concentrations of MSNs were 8.94 and 1.87 days, respectively, while for the lowest and highest concentrations of CESN were 10.02 and 5.23 days, respectively, for the lowest and highest concentrations of cinnamon oil were 31.18 and 2.93 days, respectively, also for the lowest and highest concentrations of peppermint oil were 33.53 and 8.66 days, respectively, and finally the lowest and highest concentrations of silica gel were 56.35 and 14.56 days, respectively. This indicates that MSNs is the most effective, followed by CESN and silica gel.

**Table 3:** Sub-lethal time of tested MSNs, CESN, cinnamon oil, silica gel, and peppermint oil

Substances used	Conc. mg/cm^2^	LT_50_ (days) (higher limit-lower limit)
MSNs	15	8.94 (10.23–7.82)
	30	5.63 (6.73–4.69)
	60	2.36 (2.85–1.94)
	90	1.87 (2.19–1.58)
CESN	15	10.02 (10.76–9.33)
	30	7.22 (7.85–6.64)
	60	5.84 (6.40–5.32)
	90	5.23 (5.75–4.76)
Silica gel	15	56.35 (338.58–9.77)
	30	56.50 (241.09–13.89)
	60	17.84 (23.31–13.70)
	90	14.56 (18.82–11.31)
Cinnamon oil	125	31.18 (48.43–20.37)
	250	7.82 (8.86–6.89)
	375	4.81 (5.44–4.25)
	500	2.93 (3.38–2.53)
Peppermint oil	125	33.53 (47.05–24.09)
	250	17.75 (21.10–14.97)
	375	11.30 (12.59–10.14)
	500	8.66 (9.68–7.74)

The LT_50_ values of the tested substances against 6th instar larvae of *C. cephalonica* revealed that MSNs were faster to act than CESNs and silica gel. A similar observation was obtained by Mesbah et al. (2017), who found that silica nanoparticles were more biologically active than normal silica against the Chinese beetle *Callosobruchus chinensis* (Linnaeus), where the LT_50_ values of the silica nanoparticles were 67.52, 72.23, and 83.44 h, which were less than those of normal silica at 120.15, 131.44, and 140.00 h.

In conclusion, mesoporous silica nanoparticles improved the efficiency of botanical insecticides due to their greater surface area and beneficial characteristics, such as poor water solubility and essential oil volatility, because the synthesized nanoparticles controlled their release. In fact, the application of these nanoparticles has great potential for the control of stored grain pests at low concentrations.

## Data Availability

The data used to support the findings of this study are included within the article.
